# Biodegradation of chlorpyrifos using isolates  from contaminated agricultural soil, its kinetic studies

**DOI:** 10.1038/s41598-021-88264-x

**Published:** 2021-05-14

**Authors:** Muhammad Farhan, Maqsood Ahmad, Amina Kanwal, Zahid Ali Butt, Qaiser Farid Khan, Syed Ali Raza, Haleema Qayyum, Abdul Wahid

**Affiliations:** 1 Sustainable Development Study Center, Government College University, Lahore, Pakistan; 2 Department of Environmental Sciences, Baluchistan University of Information Technology, Engineering and Management Sciences, Quetta, Pakistan; 3 Department of Botany, Government College Women University, Sialkot, Pakistan; 4 Directorate of Land Reclamation, Irrigation Department, Lahore, Pakistan; 5 Department of Chemistry, Government College University, Lahore, Pakistan; 6 Department of Environmental Science, Bahauddin Zakariya University, Multan, Pakistan

**Keywords:** Environmental sciences, Environmental microbiology

## Abstract

Extensive pesticides use is negatively disturbing the environment and humans. Pesticide bioremediation with eco-friendly techniques bears prime importance. This study evaluates the bioremediation of chlorpyrifos in soil using indigenous *Bacillus cereus* Ct3, isolated from cotton growing soils. Strains were identified through ribotyping (16s rRNA) by Macrogen (Macrogen Inc. Geumchen-gu, South Korea). *Bacillus cereus* Ct3 was resistant up to 125 mg L^−1^ of chlorpyrifos and successfully degraded 88% of chlorpyfifos in 8 days at pH 8. *Bacillus cereus* Ct3 tolerated about 30–40 °C of temperature, this is a good sign for in situ bioremediation. Green compost, farmyard manure and rice husk were tested, where ANOVA (*P* < 0.05) and Plackett–Burman design, results indicated that the farm yard manure has significant impact on degradation. It reduced the lag phase and brought maximum degradation up to 88%. Inoculum size is a statistically significant (*P* < 0.05) factor and below 10^6^ (CFU g^−1^) show lag phase of 4–6 days. Michaelis–Menten model results were as follows; R^2^ = 0.9919, V_max_ = 18.8, K_s_ = 121.4 and V_max_/K_s_ = 0.1546. GC–MS study revealed that chlorpyrifos first converted into diethylthiophosphoric acid and 3,5,6-trichloro-2-pyridinol (TCP). Later, TCP ring was broken and it was completely mineralized without any toxic byproduct. Plackett–Burman design was employed to investigate the effect of five factors. The correlation coefficient (R^2^) between experimental and predicted value is 0.94. Central composite design (CBD) was employed with design matrix of thirty one predicted and experimental values of chlorpyrifos degradation, having “lack of fit *P* value” of “0.00”. The regression coefficient obtained was R^2^ = 0.93 which indicate that the experimental vales and the predicted values are closely fitted. The most significant factors highlighted in CBD/ANOVA and surface response plots were chlorpyrifor concentration and inoculum size. *Bacillus cereus* Ct3 effectively degraded chlorpyrifos and can successfully be used for bioremediation of chlorpyrifos contaminated soils.

## Introduction

Pesticide dispersal and persistence depends on soil properties, nature of active compound and abiotic/biotic conditions^1^. In soil, pesticide fate is quite complex, they may degrade, volatilize, hydrolyze or mineralize. Soil has the tendency to restrict the movement of pesticides. Excessive use of pesticides disturbs soil microflora/fauna and as a result decreases soil fertility. Leaching of pesticide results in the ground water contamination. Studies showed that very less amount (0.1%) of applied chlorpyrifos enter the target pest, where as 99.9% disperse in environment^2^. Chlorpyrifos (CP) is “moderately toxic” (class II) broad range organophosphate insecticide with half life of 60–120 days and is used on almonds, cotton, oranges, apples and corn. It inhibit cholinesterase (AChE) in peripheral and central nervous system. In children/fetus small quantity of pesticide imparts neurotoxic effects and huge quantities exhibit severe toxicity. It may lead to lethal damage to heart and pancreas. The hydroxyl radicals volatilize in atmosphere and become more toxic in the form of chlorpyrifos-oxon. This remains in air over more than 4 h^3^. The search of new methods is in high demand to restore contaminated soils^4,5^. Bioremediation is gaining popularity due to more efficiency, eco-friendly, selective destruction and low cost^6,7^. Bioremediation has successfully decontaminated agricultural soils, wetlands, sludge and ground water^8^. Pesticides contaminated soil can be restored by injecting specific microbes^9–11^. Pesticide polluted soils are the most appropriate site to find out the resistant microbes. Native microbes develop resistance over period of time in polluted sites or soils^8,12^.

Hamzah et al*.*^10^ efficiently degraded CP by *Pseudomonas aeruginosa* in 5 days*.* Singh et al.^13^ recommended the injection of *Pseudomonas* sp. in Australian soils, this causes the fast degradation of CP. The experiment lasted for 90 days and the maximum degradation was observed in basic soils. *Enterobacter* sp. B-14 degraded 35 mg kg^−1^ of CP in soils^14^. Mixed culture proved less effective as compared to the mono-culture. For the bioremediation of diazinon *Pseudomonas* sp. was examined^15^. *Pseudomonas* showed 80–92% degradation of 100 mg K^−1^ in sterile soil in presence of glucose. The rate of degradation varies from 0.085 to 0.032 mg day^−1^ and half life varies from 25 to 11 days. Moreover, in 7 days only 2% degradation of diazinon was achieved. In statistical analysis the microbial community and biochemical processes shows positive correlation. Samuel et al.^16^ reported *Pseudomonas putida*, which has the ability to detoxify aflatoxin into non-toxic products.

Fungi are also the potential candidate for biodegradation studies in soil. *Verticillium* sp. exhibited the successful degradation of CP in different mediums. The degradation process becomes speedy by using this strain and it also competed with native microbes^17^. Similarly, 3.59 times fast degradation was reported by Fang et al.^18^. Enzymes are effective biocatalyst which can be successfully employed for environmental bioremediation^19^. Other than native strains and their enzymes, the use of genetically engineered microbes is also gaining popularity for biodegradation^20^. Current study is designed to investigate the potential of *Bacillus cereus* Ct3 to degrade CP in soil. *Bacillus cereus* Ct3 was isolated from cotton fields. Optimization of biodegradation conditions like; kinetic analysis, Plackett–Burman Design, Central Composite Design, response surface plots, ribotyping and biodegradation pathway was studied.

## Materials and methods

### Microbial strain selection, identification and Inoculum preparation

*Bacillus* strain Ct3 was selected for this study, this stain was isolated from cotton growing agricultural soils. Soil (30 g), sterile MSM (minimal salt medium), 15 mL chlorpyrifos were mixed and shaked for 1 week. After 1 week, 10 mL of this solution was transferred to freshly sterile MSM. Chlorpyrifos concentration was increased gradually up to 150 mL L^−1^. Culture was then transferred to sterile nutrient agar plates having 150 mL L^−1^ of chlorpyrifos^21^. Only resistant colonies which show growth were selected for further experimentation. Strain Ct3 showed the most prominent growth in presence of chlorpyrifos. The strain Ct3 was also tested for its resistance against other pesticides. Strains were identified through ribotyping (16s rRNA) by using reverse primer (5ʹCCGTCAATTCMTTTRAGTTT3ʹ) and universal forward (5ʹGGATTAGATACCCTGGTA3ʹ). Testing facility was provided by Macrogen (Macrogen Inc. Geumchen-gu, South Korea). The bacterial species sequences were deposited in GenBank, which showed more than 98% similarity and thus identified as *Bacillus* sp. Inoculums size was quantified by haemocytometer.

### Bio-degradation in laboratory soil and pot

Impact of concentration on bioremediation was investigated by adding sterile soil (0.1 kg), chlorpyrifos (measured quantity) and inoculum (quantified) in a sterile glass beaker. The set up was kept at desired temperature. Control was without inoculums and whole experiment was setup with three replicates. After regular intervals soil samples were tested for metabolites and residual pesticide. Effect of temperature, pH and inoculum sizes was testing using different concentration ranges. Green compost, farmyard manure and rice husk were tested as source of carbon. Measured quantity of carbon source, sterile soil, chlorpyrifos and inoculum was aseptically mixed. Similar arrangement without added carbon source was setup as control. The optimum inoculation size and chlorpyrifos concentration with other standardized conditions were tested.

### Kinetics study

Kinetic studies were carried out using Michaelis–Menten model^22^

1 $$\frac{\mathrm{dS}}{\mathrm{dt}}=-{V}_{max}\frac{\mathrm{S}}{{\mathrm{S}+K}_{s}}$$

here; ‘S’ is the ‘substrate concentration’, ‘V_max_’ is the ‘maximum biodegradation rate’ and ‘K_s_’ is the ‘half saturation constant’. Rearrangement of Eq. (1) generates “Hanes plot”, which shows straight line between [S] and [S]/v. As the experiment used intact cell instead of isolated enzymes, so we replaced the “K_s_” with “K_m_”^22^.

### Chlorpyrifos extraction and analysis

CP and its metabolites were identified by protocol of Xu et al.^23^. Petroleum ether was used to get cell-free supernatant. Supernatant was dried, redisssolved in methanol and filtered (0.45 mm pore size). Metabolites were identified by GC–MS system (Agilent, USA).

### Plackett–Burman design (PBD) for screening significant factors

In this study activity of five variables (both nutritional and environmental) were selected for statistical analysis. These variables and their higher (+ 1) and lower (− 1) values are presented in Table S5. Plackett–Burman design was designed using Minitab 16.2.2 software^24^ and the comprehensive matrix (standard 12 run) is presented in Table 1. The impact of each variable on biodegradation of chlorpyrifos was calculated through following equation:

2 $$\mathrm{Y}={\mathrm{A}}_{\mathrm{o}}+\sum {\mathrm{B}}_{\mathrm{i}}{\mathrm{X}}_{\mathrm{i}}$$

where Y represents the chlorpyrifos biodegradation (response), A_o_ represents the intercept, B_i_ represents the linear factor coefficient and X_i_ is the level of each variable. Probability value (*P* value < 0.05) in regression analysis is used to identify significant factors for chlorpyrifos biodegradation. ANOVA for PBD was generated using Minitab software. Factors were further optimized by Response surface methodology.

**Table 1:** Plackett–Burman design (PBD) of factors (in coded levels) with biodegradation of chlorpyrifos (%) as response.

Run order	Concentration	Temperature	pH	Carbon	Inoculum	Response	Predicted
1	− 1	− 1	+ 1	+ 1	+ 1	82.3	81.3
2	− 1	+ 1	+ 1	+ 1	− 1	50.3	53.7
3	+ 1	+ 1	− 1	+ 1	+ 1	72.4	62.5
4	+ 1	+ 1	+ 1	− 1	+ 1	31.8	31.2
5	− 1	− 1	− 1	+ 1	+ 1	79.3	76.5
6	+ 1	+ 1	− 1	+ 1	− 1	30.3	29.3
7	− 1	− 1	− 1	− 1	− 1	14.2	7.2
8	+ 1	− 1	− 1	− 1	+ 1	7.2	20.8
9	− 1	+ 1	+ 1	− 1	+ 1	50.7	50.7
10	+ 1	− 1	+ 1	− 1	− 1	6.3	2.3
11	− 1	+ 1	− 1	− 1	− 1	6.7	12.7
12	+ 1	− 1	+ 1	+ 1	− 1	18.4	28.7

### Central composite design (CCD) of experiments and response surface methodology (RSM)

Four significant factors viz concentration of chlorpyrifos, temperature, carbon source and inoculum size were established as critical determinants for biodegradation of chlorpyrifos as the outcome of PBD. Full factorial central composite design (CCD) (2^4^) was adopted with 31 experimental runs, seven center points and eight axial points^25^. The significance of model, prediction equation, regression coefficient and case statics were calculated through analysis of variance (ANOVA). A second-order polynomial equation was employed to fit results and analyze interaction among factors, as shown by Eq. (3):

3 $$Y={A}_{0}+\sum _{i=1}^{n}{B}_{i}{X}_{i}+ \sum _{i=1}^{n}{B}_{ii}{X}_{\mathrm{i}}^{2}+\sum _{i=1}^{n}\quad \sum _{j=1}^{n}{B}_{ij}{X}_{i}{X}_{j}$$

where ‘Y’ represents the chlorpyrifos degradation, ‘*X’* represents the factors that influence degradation, ‘*A*_*o'*_’ represents the intercept coefficient, ‘B_i_’ represents the linear coefficient, ‘B_ii_’ represents the quadratic coefficient and ‘B_ij_’ represents the interaction coefficient. Response surface plots were obtained using Minitab (statistical software, version 16.2.2) to identify effect of factors. ANOVA for CCD was generated using Minitab Software.

### Ethical approval

This article does not contain any studies with human participants or animals performed by any of the authors.

### Informed consent

Informed consent is not-applicable in this study.

## Results and discussion

**Figure 1:**
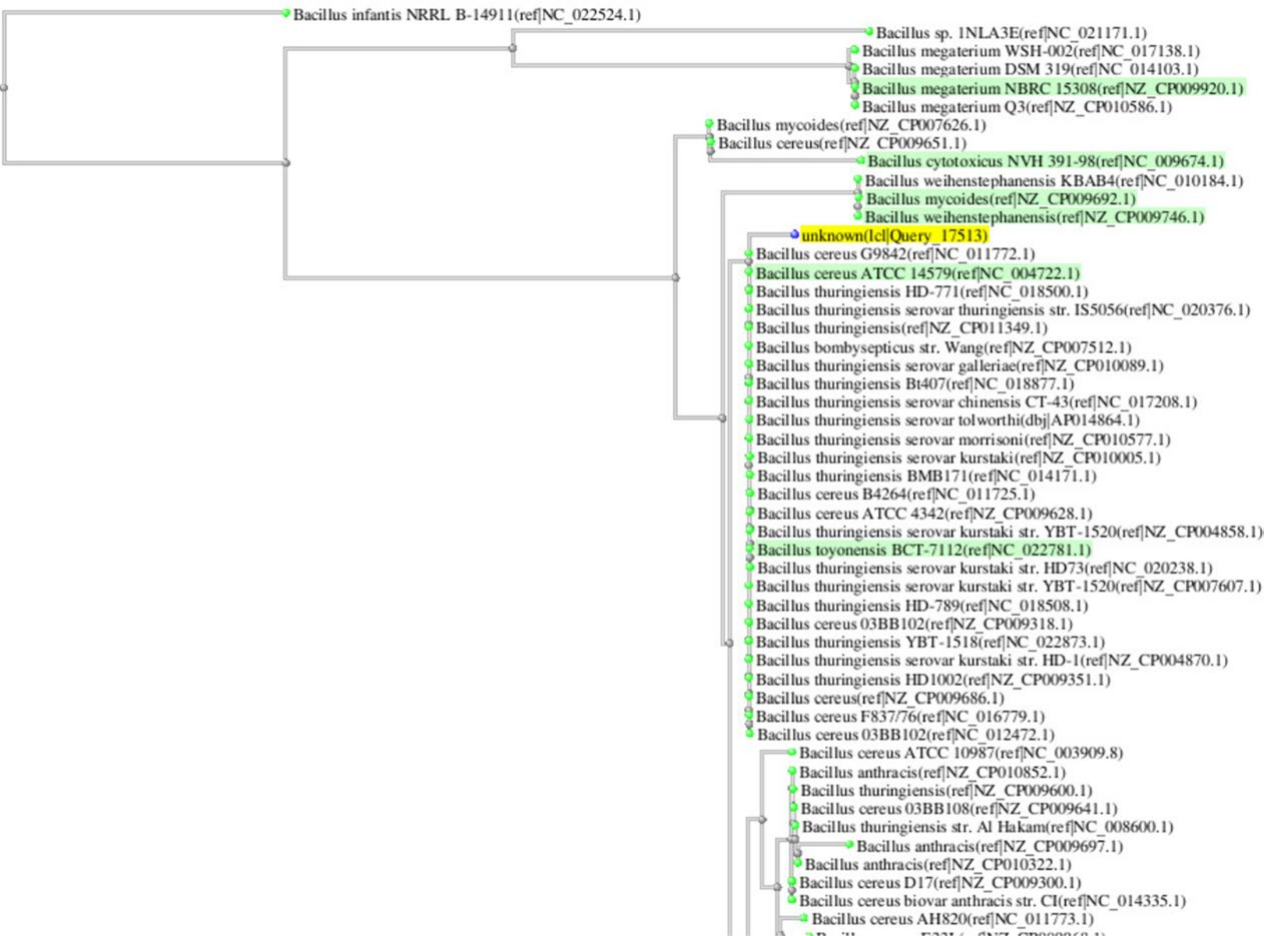
Phylogenetic tree of strain Ct3.

**Figure 2:**
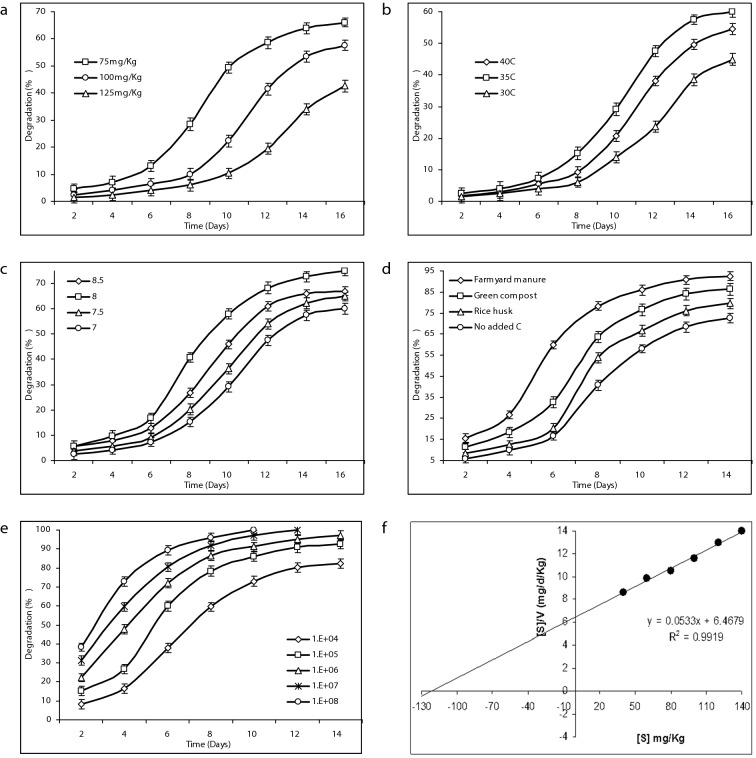
Biodegradation rate of CP at different factors in laboratory soil. (**a**) pH: 7, inoculum density: 10^5^ (CFU mL^−1^), incubation temperature: 37 °C and C-source: nil. (**b**) CP conc: 100 mg kg^−1^, pH: 7, inoculum density: 10^5^ (CFU g^−1^) and C-source: nil. (**c**) CP Conc: 100 mg kg^−1^, inoculum density: 10^5^ (CFU g^−1^), incubation temperature: 35 °C and C-source: nil. (**d**) CP Conc: 100 mg kg^−1^, inoculum density: 10^5^ (CFU g^−1^), incubation temperature: 35 °C and pH: 8. (**e**) CP Conc: 100 mg kg^−1^, pH: 8, incubation temperature: 35 °C and C-source: farmyard manure. (**f**) Hanes plot for calculating chlorpyrifos biodegradation kinetics of *Bacillus* sp. in soil.

The demand of eco-friendly solution and the use of indigenous species to restore pesticide contaminated soils is growing globally^26^. Biodegradation is considered best option for in situ restoration operations^6^. Soils contaminated with polycyclic aromatic hydrocarbon was successfully restored by *Nocardia farcinica*, Nocardia asteroids and Nocardia cashijiensis^27^. Chlorpyrifos is widely used on cotton and vegetables to control pests. Excessive use of the pesticide and its accumulation in soil changes the physiochemical properties of soil. Which lead to change in soil micro-flora and fertility. Abigail et al.^7^ reported that loss of soil fertility is due to toxicity and mutagenicity in *nostoc muscorum* and *anabaena doliolum*. To restore chlorpyrifos contaminated soil we isolated resistant strain Ct3, it was resistant up to 150 mg of chlorpyrifos. The resistance detail in different concentrations of chlorpyrifos is given in Table S1. This strain is not much resistant against other pesticides (Table S2). Its phylogenetic trees identified it as *Bacillus cereus* (Fig. 1). The use of indigenous species is preferred as they do not pose any negative impact on micro-flora. The degradation rate becomes slow at higher CP concentration, whereas at low concentration with apparently no lag phase the rate of biodegradation becomes fast (Fig. 2a). To maintain minimum number of bacteria the more time is required that becomes the reason of longer lag phase^28^. Results showed that the CP bioremediation is concentration dependent. ANOVA reveled that concentration is a significant (*P* < 0.05) factor (Tables 1, 2). 4.7% bioremediation was noted with the initial concentration of 75 mg kg^−1^ and when the concentration was increased up to 125 mg kg^−1^ the bioremediation decreased to 2% (in 2 days). After 4 days the rapid degradation started when initial concentration of 75 mg kg^−1^, whereas after 8 days of incubation, rapid degradation started when concentrations was 100 and 125 mg kg^−1^. At 16th day more degradation (66%) was noted with 75 mg kg^−1^ of CP and less degradation (43) was noted with 125 mg kg^−1^ of CP. The 2 main reasons which impact the CP degradation are as follows; firstly, as the parent pollutant (CP) concentration decreases the attractive forces between soil particles and CP molecules increases^11^. Secondly, the growth of bacterial population was hindered due to the accumulation of intermediate metabolites^29^. According to these results it is revealed that broad range of CP concentration was tolerated by the *Bacillus* sp. Hua et al.^30^ successfully degraded 12 mg kg^−1^ up to 79.5% in soil (35 days). Ahmad et al.^31^ reported resistant strain, this strain showed 97% degradation of 50 mg kg^−1^ in 45 days. This strain was identified as *Bacillus pumilus* C2A1.

**Table 2:** ANOVA for PBD model.

Source	DF	Seq SS	Adj SS	Adj MS	F	*P*
Regression	5	192.247	192.247	38.4493	69.049	0.000
Concentration	1	21.960	21.960	21.9597	39.436	0.000
Temperature	1	6.579	6.579	6.5789	11.815	0.0017437
pH	1	3.613	3.613	3.6127	6.488	0.0662283
Carbon	1	92.683	92.683	92.6833	166.444	0.0000000
Inoculum	1	67.412	67.412	67.4120	121.061	0.0000000
Error	30	16.705	16.705	0.5568		
Lack-of-fit	6	16.607	16.607	2.7678	672.568	0.0000000
Pure error	24	0.099	0.099	0.0041		
Total	35	208.952				

The failure and success of biodegradation is due to temperature variation^32^. Degradation capacity of *Bacillus* Ct3 was significantly affected temperature (*P* < 0.05) (Tables 1, 2). After 6 days rapid degradation was started (at 35 °C), whereas, lag phase continued up to 8 days at 30 °C and 40 °C. Excessive degradation of 45%, 60% and 54% was observed at 30 °C, 35 °C and 40 °C, respectively (Fig. 2b). According to the results, the isolated *Bacillus* sp tolerated wide range of temperature; this is a good sign for in situ bioremediation. Liu et al.^9^ stated that at 30 °C *Pseudomonas putida* worked most effectively and achieved 78%.

In bioremediation process, soil pH plays one of the most effective roles as abiotic factors. The CP bioremediation rate changes with small fluctuation in pH. The maximum degradation (75%) of CP was observed at 8 pH (Fig. 2c). After 6 days, the CP degradation increase sharply and later at day 14 it entered into stationary phase. Maximum degradation rate of 67% was observed at pH 8.5 and the minimum (60%) was observed at pH 7. All the applied treatments of pH showed significant difference from each other at the 16th day. Whereas, non-significant results were observed at 2nd and 4th day. Impact of pH change was non-significant (*P* < 0.05) on degradation (Table 2). Results also showed that with wide range of pH the *Bacillus* can effectively degrade CP (Fig. 2c). Other research projects also highlighted the significance of pH in bioremediation^33^. In the rapid changing environment, the strains which had the ability to work at variable pH are more likely to succeed in degradation and are more acceptable. *Enterobacter* sp. was isolated by Singh et al.^14^, this strain showed optimum degradation at higher pH, whereas low pH had negative effect on this strain. *Pseudomonas kilonensis* SRK1 was isolated from wastewater which degraded initial concentration of 150 mg L^−1^ having CFU (3.6 × 10^6^), pH (8) and glucose as additional carbon source^34^. Samual et al.^35^ reported optimum biodegradation of *p*-nitrophenol at pH 7 using *Pseudomonas putida.* Gong et al.^36^ stated that optimum pH is one of the critical factor in biodegradation and successive cycling of pollutants in forest*.*

In bioremediation process the organic amendments significantly impact the rate and course of degradation. In this study, rice husk, farmyard manure and green compost was added in the soil as carbon source. In presence of organic amendments *Bacillus* sp. showed changed behavior towards bioremediation (Fig. 2d). Farmyard manure shows more promising results towards bioremediation process in comparison to non-carbon treatment. ANOVA (*P* < 0.05) and PBD indicated that the carbon source has significant impact on degradation (Table 2). It reduced the lag phase and brought maximum degradation up to 88%. During whole experiment the degradation percentage varies significantly. The increase in degradation rate from 1st to 14th day was gradual, but degradation rate becomes slow after day 14th (Fig. 2d). With addition of green compost the degradation was also facilitated and reached to the level of 83% without lag phase. However, addition of rice husk showed different pattern. Initially lag phase was observed, later degradation increased at 6th day and reached at stationary phase at 14th day. Rice husk is 13% less effective than farmyard manure. This result shows that *Bacillus* sp. can effectively use different organic material to speed up the bioremediation process. Many studies have recommended the effectiveness of amendments especially organic one, common examples includes public green compost, farmyard manure, municipal waste, cow manure^37^, mushroom spent, nut shells^38^ and coconut fiber^39^.

In CP bioremediation the inoculum size/density also play a vital role. At different inoculum densities this strain depicts almost the similar pattern (Fig. 2e). With 10^8^ CFU g^−1^ the CP degradation was up to 100% (in 10 days). Inoculums density of 10^7^ CFU g^−1^ also led to 100% degradation. However, medium inoculums densities (10^4^ and 10^5^ CFU g m^−1^) reported slightly less degradation rate initially but later fast degradation starts. Inoculums density of 10^7^ (CFU g^−1^) also attained 100% degradation. *Cupriavidus* sp. DT-1 also degraded 100% of CP and 94% of TCP. Inoculum is a statistically significant (*P* < 0.05) factor in chlorpyrifos degradation (Table 2). The whole experiment lasted for 30 days with 10^6^ cells g^−1^ ^40^. The use of sewage sludge for improving bioaugmentation was tested and found effective^41^. Jariyal et al.^42^ has reported that instead of single strain, consortia can be used for better degradation. Use of consortium is also very beneficial in synergistic biodegradation of aromatic-aliphatic copolyester plastic. This consortium was isolated from marine ecosystem and have the tendency to use pollutant as sole carbon source in 15 days^43^.

**Figure 3:**
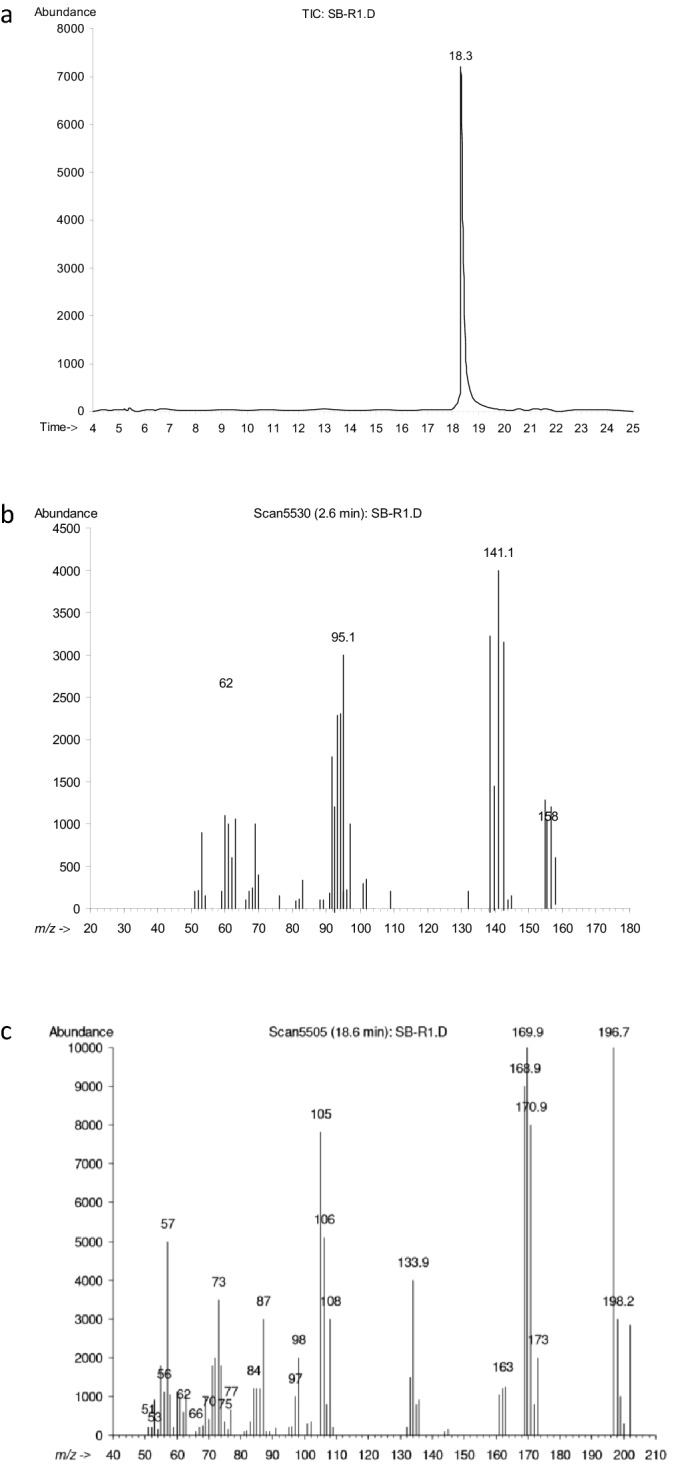
HPLC chromatograms indicating metabolites of chlorpyrifos (**a**), (**b**) & (**c**).

**Figure 4:**
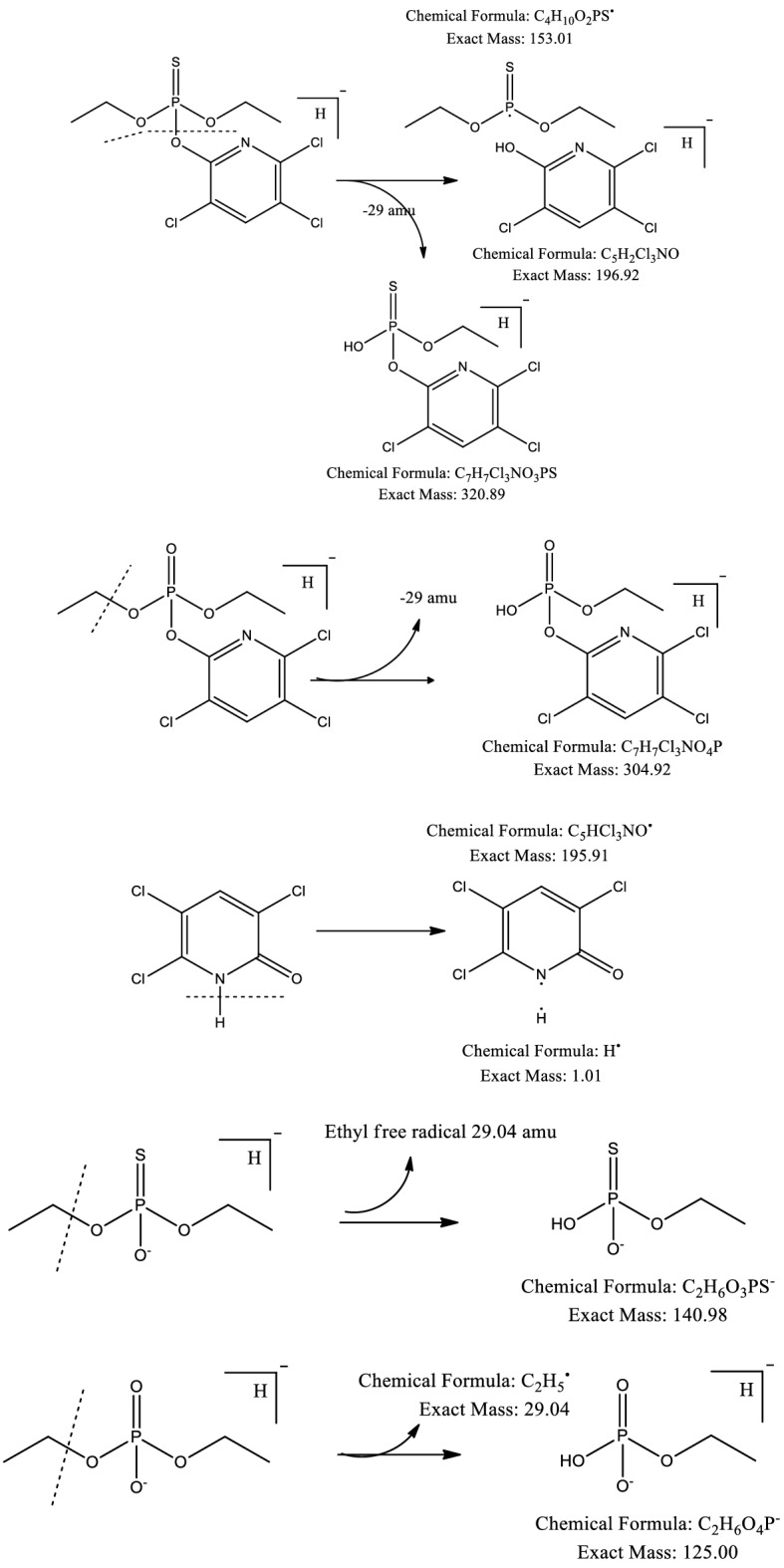
Purposed degradation pathway of chlorpyrifos.

The Hanes plot’s straightness is signified by the R^2^ (0.9919) and V_max_ (18.7627) (Fig. 2f)*.* Effectiveness of the isolates is usually predicted by the ratio of V_max_/K_s,_ and in this study this ratio is 0.1546. Maya et al.^22^ investigated the degradation of CP and TCP with the addition of *Pseudomonas, Bacillus* and *Agrobacterium *sp. K_m_ varies from 97 to 142.3 mg L^−1^ for CP and for TCP K_m_ range was 103.09–148.8 mg L^−1^. The range of V_max_ of CP was from 7.4 to 12.1 mg L^−1^. Whereas, V_max_ of TCP range from 14.90 to 21.20 mg L^−1^. Fang et al.^18^ used *Verticillium* sp. for bioremediation of CP and calculated V_max_ = 12.2 and R^2^ = 0.98. Geed et al.^29^ calculated bioremediation kinetics of Malathion (pesticide) under inhibitory and non-inhibitory conditions. The kinetic constants were calculated by using Monod models and the values were as follows, Ks = 126.30 mg L^−1^ and μmax = 0.27 day^−1^. Samuel et al.^44^ reported that pseudo-secound order model to be best fit for Pb(II) removal. The kinetic data was as follows, R^2^ = 0.987, q_e_ = 24.64, K_2_ (min^−1^) = 0.0342 and q_max_ (mg g^−1^) = 112.35. In GC–MS chromatogram, 2 peaks appeared at retention time (RT) of 9.14 and 15.13 min (Fig. 3). These peaks were designated “A” and “B”. Peak “B” with retention time 15.13 min match with the chlorpyrifos. With the passage of time this peak decreased gradually. Peak “A” with the RT of 9.14 min match with TCP (Fig. 3). Characteristic fragment ion peak and molecular ion (*m*/*z*) also supported the identification of TCP. Peak “A” was most prominent around 7–15 days, later this peak started decreasing. The CP first converted into diethylthiophosphoric acid (DETP, *m*/*z* = 172) and TCP (*m*/*z* = 197) by hydrolysis (Table S3). Figure 4 presents the proposed degradation pathway of chlorpyrifos. The TCP ring was broken and it was completely mineralized without any accumulation of byproduct. The degradation products were non-toxic as they did not hinder the growth of *Bacillus cereus* Ct3. Very good growth was noted till 14th day of biodegradation experiment (Table S4). Similar pathway and metabolites were reported by other studies^22,45^. Hamzah et al.^10^ reported 3,5,6-trichloro-2-pyridinol and CPF-oxon as metabolite of CP degradation by *Pseudomonas aeruginosa.* The metabolites identified in this study were diethyl phosphate and 3,5,6-trichloro-2-pyridinol, these 2 metabolites were also reported by Liu et al*.*^9^. Samuel et al.^46^ calculated q_max_ = 71.9 and R^2^ = 0.99 for biosorption of chromium by *Aspergillus niger*. Table 3 reports the chlorpyrifos degradation efficiencies of different strains in different conditions.

**Table 3:** Comparison of chlorpyrifos biodegradation efficiencies.

Study	Isolate used	Degradation (%)	Initial concentration (mg L^−^^1^)	Time to degrade (days)
Present study	*Bacillus cereus *Ct3	87	125	8
Chishti et al.^47^	*Enterobacter *sp. SWLC2	87	100	18
Soares et al.^48^	*Aspergillus sydowii *CBMAI 935	32	192	10
Jin et al.^49^	*Trametes versicolor*	90	150	8
Gangola et al.^50^	*Bacillus subtilis*	95	200	15
Gao et al.^51^	*Cladosporium cladosporioides *Hu*-*01	>90	50	5
Kulshrestha and Kumari^52^	*Acremonium* sp. (GFRC*-*1)	84	300	20
Zhu et al.^53^	*Bacillus* *licheniformis *ZHU-1	100	100	14
Anwar et al.^54^	*Bacillus* *pumilus* C2A1	89	1000	15
Korade and Fulekar^55^	*Pseudomonas nitroreducens* PS-2	100	100	28
Fang et al.^56^	*Verticillium* sp. DSP	100	100	7
Li et al.^57^	*Sphingomonas* sp.	98	30	10

Plackett–Burman design was employed to investigate the effect of five factors. Table 1 represents the experimental design with the actual chlorpyrifos degradation values and the predicted chlorpyrifos degradation values. The correlation coefficient (R^2^) between experimental and predicted value is 0.94, which is in reasonable agreement. Table 2 represents the ANOVA results for PBD, where *P* value less than 0.05 represents the significance of the factor for chlorpyrifos biodegradation. The polynomial equation for independent and dependent factors in this study could be written as:

4 $$\begin{aligned} {\text{Y }} & = \, - 0.51 \, {-} \, 0.0313*{\text{concentration }} + \, 0.09*{\text{temperature}} \\ & \quad + \, 0.422{\text{pH }} + \, 1.07*{\text{carbon }} + \, 2.737*{\text{Inoculum}}{.} \\ \end{aligned}$$

Base on the results of PBD following four significant factors were identified; concentration, temperature, carbon, inoculum size. Central composite design (CBD) was employed and its design matrix with thirty one predicted and experimental values of chlorpyrifos degradation is represented in Table 4. The second-degree polynomial equation obtained was as follows:

5 $$\begin{aligned} {\text{Y }} & = \, 31.22 \, + \, 0.027*{\text{concentration }} + \, 2.057*{\text{temperature}} \\ & \quad + \, 1.456*{\text{carbon }} + \, 5.656*{\text{inoculum }}{-} \, 0.0003\,{\text{concentration}}*{\text{concentration}} \\ & \quad {-} \, 0.029{\text{temperature}}*{\text{temperature }}{-} \, 0.37{\text{carbon}}*{\text{carbon}} \\ & \quad {-} \, 3.357{\text{inoculum}}*{\text{inoculum}}{.} \\ \end{aligned}$$

**Figure 5:**
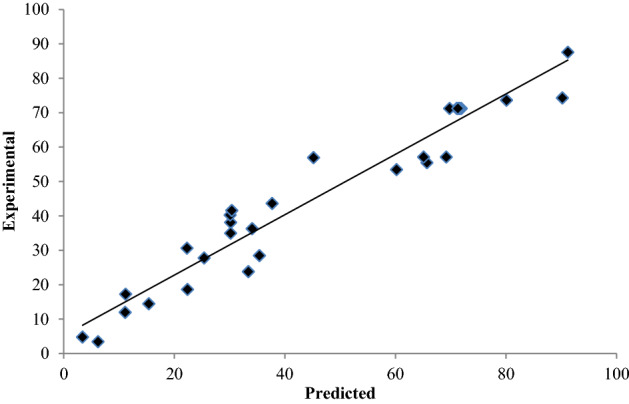
The predicted values plotted against actual values derived from CCD model for chlorpyrifos biodegradation.

**Figure 6:**
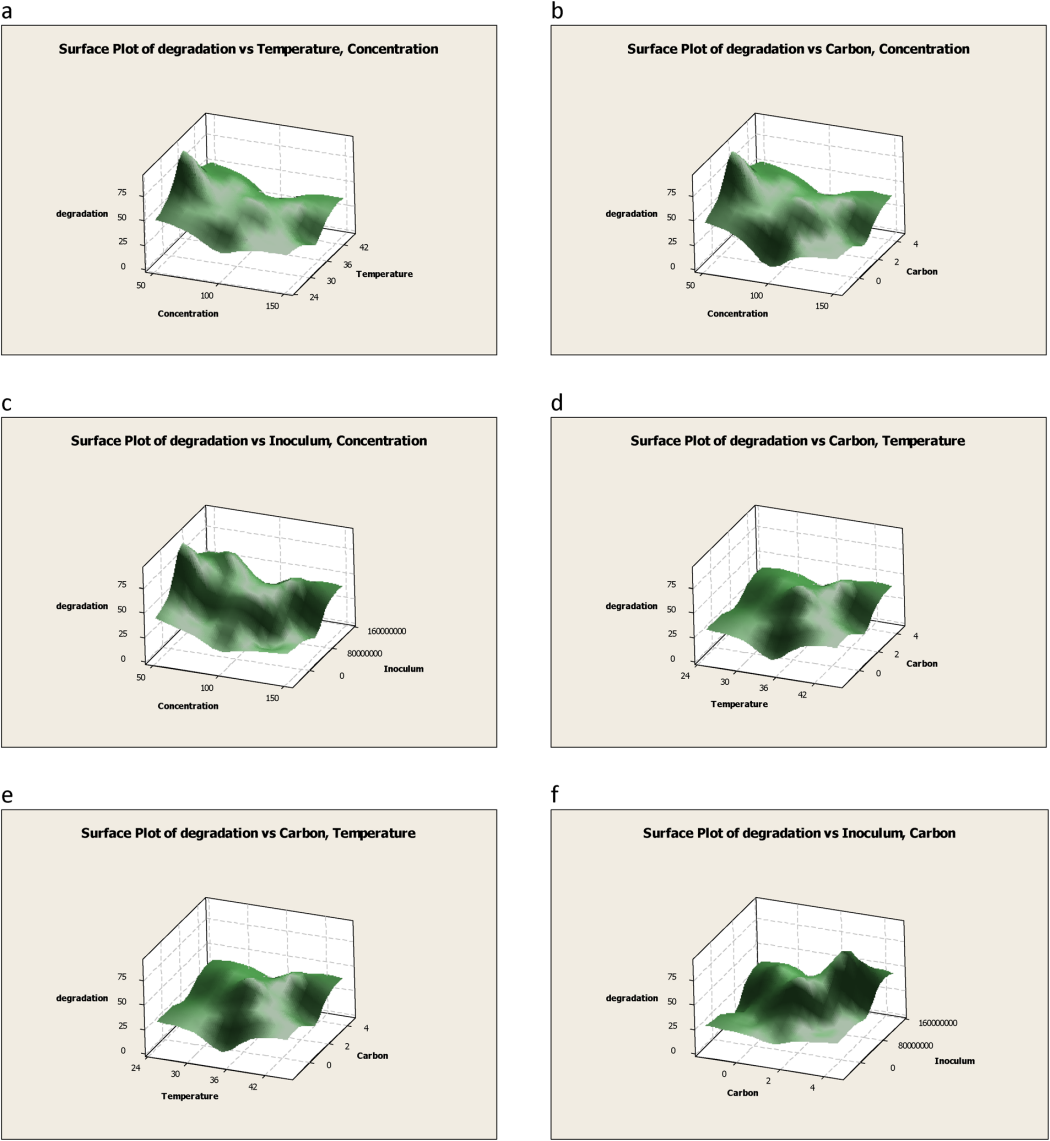
Surface plot showing interaction between different variables. (**a**) Interaction between degradation, temperature and chlorpyrifos concentration. (**b**) Interaction between degradation, carbon source and chlorpyrifos concentration. (**c**) Interaction between degradation, inoculum size and chlorpyrifos concentration. (**d**) Interaction between degradation, carbon source and temperature. (**e**) Interaction between degradation, carbon source and temperature. (**f**) Interaction between degradation, inoculum size and carbon source.

The ANOVA result for CBD is presented in Table 5. It represents the individual main effects and the interaction among different factors. The “F-value” of the model was 1050.7 which suggest that the model was significant. For the present study the “lack of fit *P* value” was 0.00. Figure 5 represents the experimental values and the predicted values for the biodegradation of chlorpyrifos. The regression coefficient obtained was R^2^ = 0.93 which indicate that the experimental vales and the predicted values are closely fitted. Figure 6a–f represents the combined effect of different factors for biodegradation of chlorpyrifos. The degradation increase with the increase in concentration, temperature, carbon source and inoculum but at certain point it reaches the maximum. Further increase in any factor beyond this point will decrease the degradation of chlorpyrifos. The most significant factors highlighted in ANOVA and surface response plots were concentration and inoculum size. Changes in concentration and inoculum size impacts more on degradation then other factors. This is in argument with the work reported by Zhou et al.^58^ and Ungureanu et al.^59^, wherein the inoculum size was the important factor for phenol degradation. Samuel et al.^60^ concluded (using response surface methodology) that biosorbent by *Certocystis paradoxa* MSR2 depends on temperature, pH and initial concentration. Similarly, Alice et al.^61^ optimized biosorption of chromium based on response surface method. Variables optimized were contact time, initial concentration and biosorbent dose.

**Table 4:** Experimental design and results of central composite design (CCD).

Run order	Concentration	Temperature	Carbon	Inoculum	Response	
					Experimental	Predicted
1	100	35	1.5	50005000	71.1	71.2
2	100	45	1.5	50005000	30.2	38.1
3	75	30	3	100000000	80.1	73.6
4	125	40	3	10000	22.4	18.6
5	50	35	1.5	50005000	91.2	87.6
6	75	30	3	10000	22.3	30.6
7	125	40	0	10000	15.4	14.5
8	100	35	− 1.5	50005000	11.2	17.3
9	125	40	0	100000000	35.4	28.5
10	100	35	1.5	50005000	71.5	71.2
11	75	40	0	10000	37.7	43.6
12	100	35	1.5	50005000	71.9	71.2
13	125	30	3	10000	11.1	12.0
14	125	30	3	100000000	65.7	55.5
15	125	40	3	100000000	69.2	57.1
16	100	35	1.5	50005000	69.8	71.2
17	75	40	0	100000000	65.1	57.1
18	125	30	0	100000000	33.4	23.8
19	100	35	1.5	50005000	71.6	71.2
20	75	40	3	100000000	90.2	74.3
21	100	25	1.5	50005000	25.4	27.8
22	100	35	1.5	− 49985000	6.2	3.4
23	75	40	3	10000	34.1	36.3
24	150	35	1.5	50005000	30.1	40.3
25	100	35	1.5	149995000	45.2	56.9
26	125	30	0	10000	3.4	4.8
27	100	35	1.5	50005000	71.5	71.2
28	75	30	0	100000000	60.2	53.5
29	75	30	0	10000	30.2	35.0
30	100	35	4.5	50005000	30.4	41.6
31	100	35	1.5	50005000	71.3	71.2

**Table 5:** ANOVA for central composite design model (CCD).

Source	DF	Adj SS	Adj MS	F	*P*
Regression	14	17,523.8	1251.70	4.09	0.004
**Linear**	4	9282.5	2320.63	7.58	0.001
Concentration	1	3360.7	3360.67	10.97	0.004
Temperature	1	160.2	160.17	0.52	0.480
Carbon	1	888.2	888.17	2.90	0.108
Inoculum	1	4873.5	4873.50	15.91	0.001
**Square**	4	7473.6	1868.39	6.10	0.004
Concentration * concentration	1	95.9	95.93	0.31	0.584
Temperature * temperature	1	2625.3	2625.26	8.57	0.010
Carbon * carbon	1	3126.6	3126.64	10.21	0.006
Inoculum * inoculum	1	3277.9	3277.93	10.70	0.005
**Interaction**	6	767.8	127.96	0.42	0.857
Concentration * temperature	1	1.0	1.00	0.00	0.955
Concentration * carbon	1	132.2	132.25	0.43	0.520
Concentration * inoculum	1	0.2	0.25	0.00	0.978
Temperature * carbon	1	9.0	9.00	0.03	0.866
Temperature * inoculum	1	25.0	25.00	0.08	0.779
Carbon * inoculum	1	600.3	600.25	1.96	0.181
**Residual error**	16	4901.4	306.34		
Lack-of-fit	10	4898.6	489.86	1050.7	0.000
Pure error	6	2.8	0.47		
Total	30	22,425.2

## Conclusion

Rapid increase in pesticide is negatively linked with ecological disturbance, decrease in soil fertility and changes in microbial community. Natural and eco-friendly techniques are gaining popularity to protect ecosystems from the side effects of pesticides. To restore chlorpyrifos contaminated soil, we isolated 156 strains from cotton growing Punjab region of Pakistan. *Bacillus cereus* Ct3 was most resistant up to 175 mg L^−1^ of chlorpyrifos. The use of indigenous specie is preferred as they do not pose any negative impact on micro-flora and has better survival chance. The process of biodegradation was optimized using Michaelis–Menten model and 88% degradation was achieved in 8 days at pH 8. Biodegradation pathway was proposed, where CP first degraded into diethylthiophosphoric acid and 3,5,6-trichloro-2-pyridinol (by hydrolysis, TCP further mineralized. Plackett–Burman design, Central composite design and surface response methods was employed to investigate the effect of five factors. Base on the results significant factors identified were chlorpyrifos concentration and inoculum size. *Bacillus cereus* Ct3 can be employed to degraded chlorpyrifos without producing toxic metabolite and can successfully be used for bioremediation of chlorpyrifos contaminated soils. Future study will focus on the whole genome sequencing of *Bacillus cereus* Ct3, identification of genes involved in biodegradation and action of enzymes responsible.

## Supplementary information

Supplementary Tables.
