# Supplementary material for: Biodegradation of chlorpyrifos using isolates from contaminated agricultural soil, its kinetic studies
Source: Sci Rep. 2021 May 14;11:10320. doi: 10.1038/s41598-021-88264-x (PMC8121937; doi:10.1038/s41598-021-88264-x)
Supplement: Supplementary file 1 — Supplementary Tables. [file 41598_2021_88264_MOESM1_ESM.doc]

**Supplementary Material for**

**Biodegradation of chlorpyrifos using isolates form contaminated agricultural soil, its kinetic studies**

Muhammad Farhan^1,*^, Maqsood Ahmad^2^, Amina Kanwal^3^, Zahid Ali Butt^3^, Qaiser Farid Khan^1^, Syed Ali Raza ^4,5^, Haleema Qayyum^1^, Abdul Wahid^6^

^1^Sustainable Development Study Center, Government College University, Lahore, Pakistan

^2^Department of Environmental Sciences, Baluchistan University of Information Technology, Engineering and Management Sciences, Quetta, Pakistan

^3^Department of Botany, Government College Women University, Sialkot, Pakistan

^4^Directorate of Soil Reclamation, Irrigation Department, Government of the Punjab, Pakistan

^5^Department of Chemistry, Government College University, Lahore, Pakistan

^6^Department of Environmental Science, Bahauddin Zakariya University, Multan, Pakistan

^*^Email; m.farhan_gcu@yahoo.com, Cell #; +92 321 4122078, ORCID; https://orcid.org/0000-0002-1509-9723

**Table S1. Screening of isolates from soil of cotton fields on chlorpyrifos amended medium**

| **Isolates** | **Area** | **Chlorpyrifos concentration (mgL^-1^)**  **50 75 100 125 150 175** | | | | | |
| --- | --- | --- | --- | --- | --- | --- | --- |
| **Ct 1** | Multan | + + + | + + | - | - | - | - |
| **Ct 2** | Multan | + + | + + | + | - | - | - |
| **Ct 3** | Multan | + + + | + + + | + + + | + + + | + | + |
| **Ct 4** | Multan | + + + | + | + | - | - | - |
| **Ct 5** | Multan | + + + | + | - | - | - | - |
| **Ct 6** | Multan | + + + | + + + | + + + | + ++ | - | - |
| **Ct 7** | Multan | + + + | + + | + | - | - | - |
| **Ct 8** | Multan | + + + | + + | + + | + | - | - |
| **Ct 9** | Multan | + + | + | - | - | - | - |
| **Ct 11** | Multan | + + + | + + | + + | + | - | - |
| **Ct 12** | Multan | + + + | + | + | - | - | - |
| **Ct 13** | Multan | + + + | + | + | - | - | - |
| **Ct 14** | Multan | + + + | + | - | - | - | - |
| **Ct 15** | Multan | + + + | + | - | - | - | - |
| **Ct 16** | Multan | + | + | - | - | - | - |
| **Ct 17** | Multan | + + | + | - | - | - | - |
| **Ct 18** | Multan | + + + | + + + | + + | + | - | - |
| **Ct 19** | Multan | + + + | + | + | - | - | - |
| **Ct 20** | Multan | + + + | + + | + | - | - | - |
| **Ct 21** | Multan | + | - | - | - | - | - |
| **Ct 22** | Multan | + + + | + | - | - | - | - |
| **Ct 23** | Multan | + + | + | - | - | - | - |
| **Ct 24** | Bahawalpur | + + | + + | + | - | - | - |
| **Ct 25** | Bahawalpur | + + + | + + + | + + | + | - | - |
| **Ct 26** | Bahawalpur | + + + | + + | + | - | - | - |
| **Ct 29** | Bahawalpur | + + | + | - | - | - | - |
| **Ct 30** | Bahawalpur | + | + | - | - | - | - |
| **Ct 31** | Bahawalpur | + + | + + | + | - | - | - |
| **Ct 32** | Bahawalpur | + + + | + + + | + + | + | - | - |
| **Ct 33** | Bahawalpur | + + + | + + | + | - | - | - |
| **Ct 34** | Bahawalpur | + + | + + | + | - | - | - |
| **Ct 35** | Bahawalpur | + + | + + | + | + | - | - |
| **Ct 36** | Bahawalpur | + | + | - | - | - | - |
| **Ct 37** | Bahawalpur | + | - | - | - | - | - |
| **Ct 38** | Bahawalpur | + + | + + | + | + | - | - |
| **Ct 39** | Bahawalpur | + + + | + + + | + + | + | - | - |
| **Ct 41** | Bahawalnagar | + + + | + + + | + + | - | - | - |
| **Ct 42** | Bahawalnagar | + + + | + + + | + + | + | - | - |
| **Ct 43** | Bahawalnagar | + + + | + | + | - | - | - |
| **Ct 44** | Bahawalnagar | + + | + + | + | - | - | - |
| **Ct 45** | Bahawalnagar | + + | + + | - | - | - | - |
| **Ct 46** | Bahawalnagar | + + | + | - | - | - | - |
| **Ct 47** | Bahawalnagar | + + + | + + + | + + | - | - | - |
| **Ct 48** | Rahim Yar Khan | + + | + + | + | + | - | - |
| **Ct 49** | Rahim Yar Khan | + | + | - | - | - | - |
| **Ct 50** | Rahim Yar Khan | + + | + | - | - | - | - |
| **Ct 51** | Rahim Yar Khan | + + | + + | + | + | - | - |
| **Ct 52** | Rahim Yar Khan | + + | + | - | - | - | - |
| **Ct 53** | Rahim Yar Khan | + + | + + | + | - | - | - |
| **Ct 54** | Rahim Yar Khan | + + | + + | + | + | - | - |
| **Ct 55** | Rahim Yar Khan | + + + | + + + | + + | + | - | - |
| **Ct 56** | Rahim Yar Khan | + + + | + + | + | + | - | - |

+ + + “very good growth”

+ + “moderate growth”

+ “poor growth”

- “no growth”

**Table S2. Screening of Ct3 isolate for different organophosphate pesticides amended medium**

| **Pesticides types** | **Pesticides concentration (mgL^-1^)** | | | | | |
| --- | --- | --- | --- | --- | --- | --- |
|  | **5** | **7** | **9** | **11** | **13** | **15** |
| **Triazophos** | ++ | + | + | - | - | - |
| **Profenofos** | + | - | - | - | - | - |
| **Fenitrothion** | ++ | + | - | - | - | - |
| **Parathion** | + | - | - | - | - | - |
| **Diazinon** | + | + | - | - | - | - |

+ + + “very good growth”

+ + “moderate growth”

+ “poor growth”

- “no growth”

**Table S3 Summary of mass spectrometric data**

| **Sr. #** | **Retention time (min)** | **Product**  **(*m/z*)** | **Product ion**  **(*m/z*)** | **Purposed compound** | **Purposed structure** |
| --- | --- | --- | --- | --- | --- |
| 1 | 16.3 | 350 | 322, 294,198 | Chlorpyrifor (CP) | 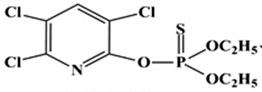 |
| 2 | 12.65 | 334 | 306, 278 | Chlorpyrifos-oxon (CPO) | 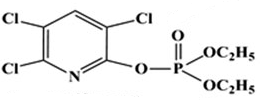 |
| 3. | 10.02 | 196 |  | 3 ,5 ,6-Trichloropyridin-2-ol (TCP) | 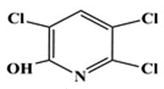 |
| 4. | 2.36 | 170 | 141, 95 | Diethyl thiophosphate (DETP) | 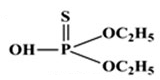 |
| 5 | <1.0 | 153 | 125, 79 | Diethyl phosphate | 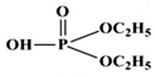 |

**Table S4. Biodegradation of chlorpyrifos in pot experiment under optimum conditions**

| **Experimental conditions** | **Parameters** | **Time (days)** | | | | | | |
| --- | --- | --- | --- | --- | --- | --- | --- | --- |
|  |  | **2** | **4** | **6** | **8** | **10** | **12** | **14** |
| CP = 100mgKg^-1^  Temp = 35^o^C  Carbon source = farm manure  Inoculum = 107 CFU | Survival of Bt3 | + | ++ | ++ | +++ | +++ | +++ | +++ |
|  | Chlorpyrifos biodegradation (%) | ND | 4 | 9 | 16 | 23 | 34 | 44 |

+ + + “very good growth”

+ + “moderate growth”

+ “poor growth”

- “no growth”

ND “not detected”

**Table S5. Selected factors with lower and higher experimental values**

| **Factors** | **Lower (-1)value** | **Higher (+1) value** |
| --- | --- | --- |
| Concentration (mgL^-1^) | 75 | 125 |
| Temperature (^o^C) | 30 | 40 |
| pH | 7 | 8.5 |
| Carbon source | 0 | 3 |
| Inoculum size (CFU/ml) | 10^4^ | 10^8^ |
